# Dr. Achilles Rose

**Published:** 1916-03

**Authors:** 


### OBITUARY

Readers are requested to report promptly the death of all physicians in Western New York, or former residents of this region, or graduates of any medical school in Western New York, and to notify the families of the deceased of our desire to publish adequate Obituary notices.

**Dr. Achilles Rose,** P. & S. (Columbia) 1872, born in Ohrdruf, near Gotha, Germany, Nov. 4, 1839, died in New York Jan. 10, of pneumonia. He was educated at Zurich and Jena. leaving the latter in 1863 and coming to this country. He entered the U. S. Volunteers and served in 1864 and 1865. As a practitioner, his most important work was along the lines of external abdominal support for splanchnoptosis, and of balneology, including continuous immersion in typhoid and the use of the carbon dioxid bath and enema. A few years ago, we reviewed his book on Napoleon's Campaign in Russia, and he had written several articles dealing with medical history, especially along military lines. He was best known as an ardent advocate of reform of medical onamatology, implying that he was one of the most thorough students of ancient and modern Greek in the world. He had been decorated by the King of Greece and made an honorary member of the Academy of Medicine of Athens. The time is not ripe to pass judgement on this phase of his life work. Some hold that a mongrel vocabulary has the same advantages as mongrel blood. Others believe that, with greater general education of the people, and especially of the various professions with which ours comes into comparison, the prolific formation of technical terms of mixed Greek and Latin origin, will be a cause of shame to the medical profession and will even detract from its influence. This journal has always supported Dr. Rose in his general contentions, but has hesitated to supplant well established terms with those of pure ancestry but unfamiliar. We understand that Dr. Rose had, in preparation, a medical lexicon of correct Greek derivatives. This will be a worthy monument to Dr. Rose's medico-literary studies, even if it remains nothing but a curiosity of some library. It is altogether possible that it may be the foundation of a revolutionary change in our professional vocabulary. Only the future can tell. At any rate, it is fitting, even in these days of tolerance and freedom from prejudice, to recognize that the patient, persevering life work which Dr. Rose performed, almost without support and usually in the face of lack of appreciation, even of a certain kind of hostility, constitutes a form of heroism.

